# Unraveling the genetic basis of *Rhizobium rhizogenes*-mediated transformation and hairy root formation in rose using a genome-wide association study

**DOI:** 10.1007/s00299-024-03388-4

**Published:** 2024-12-03

**Authors:** Philipp Rüter, Thomas Debener, Traud Winkelmann

**Affiliations:** 1 Institute of Horticultural Production Systems, Section Woody Plant and Propagation Physiology, Leibniz University Hannover, Herrenhäuser Str. 2, 30419 Hannover, Germany; 2 Institute of Plant Genetics, Section Molecular Plant Breeding, Leibniz University Hannover, Herrenhäuser Str. 2, 30419 Hannover, Germany

**Keywords:** *Agrobacterium rhizogenes*, Adventitious root formation, Callus formation, GWAS, *Rosa* cultivars, SNP marker

## Abstract

**Key Message:**

Multiple QTLs reveal the polygenic nature of *R. rhizogenes*-mediated transformation and hairy root formation in roses, with five key regions explaining 12.0–26.9% of trait variability and transformation-related candidate genes identified.

**Abstract:**

Understanding genetic mechanisms of plant transformation remains crucial for biotechnology. This is particularly relevant for roses and other woody ornamentals that exhibit recalcitrant behavior in transformation procedures. *Rhizobium rhizogenes*-mediated transformation leading to hairy root (HR) formation provides an excellent model system to study transformation processes and host–pathogen interactions. Therefore, this study aimed to identify quantitative trait loci (QTLs) associated with HR formation and explore their relationship with adventitious root (AR) formation in rose as a model for woody ornamentals. A diversity panel of 104 in vitro grown rose genotypes was transformed with *R. rhizogenes* strain ATCC 15834 carrying a green fluorescent protein reporter gene. Phenotypic data on callus and root formation were collected for laminae and petioles. A genome-wide association study using 23,419 single-nucleotide polymorphism markers revealed significant QTLs on chromosomes one and two for root formation traits. Five key genomic regions explained 12.0–26.9% of trait variability, with some peaks overlapping previously reported QTLs for AR formation. This genetic overlap was supported by weak to moderate correlations between HR and AR formation traits, particularly in petioles. Candidate gene identification through literature review and transcriptomic data analysis revealed ten candidate genes involved in bacterial response, hormone signaling, and stress responses. Our findings provide new insights into the genetic control of HR formation in roses and highlight potential targets for improving transformation efficiency in ornamental crops, thereby facilitating future research and breeding applications.

**Supplementary Information:**

The online version contains supplementary material available at 10.1007/s00299-024-03388-4.

## Introduction

Plant genetic transformation allows the introduction of foreign DNA into plant cells. This process is essential for breeding programs and functional genetic studies. Transformation is most commonly achieved using bacteria of the genus *Rhizobium* (syn. *Agrobacterium*). However, effective transformation of a wide range of genotypes is often hindered by plant genetic constraints. These limitations may be imposed by the genotypes used in the transformation process. For example, certain plant species or varieties may have inherent genetic characteristics that make them more recalcitrant to transformation and/or regeneration. While many agrobacterial genes involved in pathogen–host interaction have been well studied (Tzfira and Citovsky 2006), the genetic mechanisms and interactive processes within the host genome are still being investigated today. This includes regulatory elements and proteins that play a role during the transformation process, such as pathogen attachment, recognition, defense responses, T-DNA transfer, trafficking through the cell cytoplasm, integration of T-DNA into the host genome, and finally the expression of T-DNA genes (Gelvin 2010, 2017; Lacroix and Citovsky 2013). For example, it remains largely unclear which proteins are important for T-DNA integration and which repair pathways are used for integration (Willig et al. 2018; Gelvin 2021).

### The complexity of transformation and hairy root formation

Transformation efficiency in plants is influenced by tissue characteristics and growth conditions. Explants from in vitro, greenhouse, or field conditions may show varying responses due to differences in tissue structure, plant hormone homeostasis, and physiological status (Švábová et al. 2005; Mangena et al. 2017). The transformation process begins when *Rhizobium* bacteria recognize plant-derived signals, particularly phenolic compounds such as acetosyringone, which activate virulence (*vir*) genes (Subramoni et al. 2014). These *vir* genes orchestrate the processing and preparation of T-DNA of the bacterial tumor (Ti, *R. radiobacter*) or root (Ri, *R. rhizogenes*) inducing plasmid. The host genotype can influence this process through varying levels of signaling compounds, with some genotypes even producing inhibitory substances (Zhang et al. 2000). Upon bacterial invasion, plants mount defense responses that vary by genotype. This involves recognition of PAMPs (pathogen-associated molecular patterns) and effector-triggered immunity (Pitzschke 2013). If bacteria overcome these defenses, T-DNA is transported into the plant cell nucleus through a type IV secretion system. The integration process comprises multiple host factors, including histone acetyltransferase complexes and DNA repair mechanisms (Gelvin 2021). Gene expression studies have identified various plant genes involved in this process, affecting DNA repair, metabolism, and plant hormone pathways (Willig et al. 2018).

*R. rhizogenes* transforms plant tissues by introducing T-DNA containing *rol* (*root oncogenic locus*) genes, which alter the hormonal and metabolic balance to induce hairy root (HR) formation (Tomilov et al. 2007). These *rol* genes (*rolA*-*D*) increase tissue sensitivity to auxins and stimulate secondary metabolism, leading to elevated antioxidant compound levels (Ozyigit et al. 2013; Veremeichik et al. 2016; Shkryl et al. 2022). The genetic elements influencing HR formation are likely to overlap with those involved in natural adventitious root (AR) formation. AR development in cuttings progresses through distinct phases: dedifferentiation of founder cells, hormonal induction, root primordia initiation, and final root emergence and establishing a vascular connection (De Klerk et al. 1999; Bellini et al. 2014; Druege et al. 2019).

### GWAS for identification of QTLs involved in transformation and HR formation

To identify genomic regions responsible for transformation and HR induction, a genome-wide association study (GWAS) may be a useful approach. By statistically associating SNP marker data with phenotypic data from a diverse population set, genomic regions can be identified, that have a relevant effect on the phenotypic traits under investigation. The present study aimed to identify SNPs and genomic regions associated with *R. rhizogenes*-mediated transformation and HR formation in a diversity panel of 104 in vitro grown rose genotypes. Roses were chosen, first because the relationships between HR and AR formation can be investigated, as the same genotypes have already been used in GWASs for traits related to shoot, callus, and root formation (Nguyen et al. 2017, 2020a, b; Wamhoff et al. 2023a, b). Second, in roses, the study of transformation-relevant QTLs (quantitative trait locus/i) may be crucial for improving ornamental or agronomic traits, such as susceptibility and resistance mechanisms (Gordon et al. 2024). These insights could also support breeding new cultivars through the introduction of T-DNA from wild-type *R. rhizogenes* strains to improve root systems of rootstock genotypes (Desmet et al. 2020). Since roses are considered as difficult-to-transform, information is needed on genotypic differences and possible causes of constraints to improve transformation efficiencies.

The objectives of this study were: (1) To identify QTLs controlling HR and callus formation through GWAS analysis and to evaluate their phenotypic effects; (2) To analyze the relationship between HR and AR formation by correlating HR formation traits with previously studied AR formation traits and comparison of QTL positions; (3) Identify potential candidate genes (CG) within the identified QTL regions that may regulate transformation and HR development. To achieve these objectives, a diversity panel of 104 in vitro grown rose genotypes was transformed with *R. rhizogenes* strain ATCC 15834 carrying a green fluorescent protein reporter gene.

## Materials and methods

### Plant tissue, transformation and phenotyping of HR formation

One hundred and four rose genotypes from two market segments, namely cut (63) and garden (41) roses, were propagated via axillary shoot formation in vitro and 30 leaf explants were transformed with *R. rhizogenes* strain ATCC 15834 (Hopkins and Durbin 1971), kindly provided by Dr. Frank Dunemann, Julius Kühn Institute, Quedlinburg, Germany, carrying a E*GFP* reporter gene with an intron under control of a ubiquitin promotor. The plasmid C757pGFPU10-35 s-ocs-LH was obtained from DNA Cloning Service, Hamburg, Germany. Each genotype was transformed twice and each transformation experiment involved between 8 and 20 genotypes. The transformation protocol was described in detail by Rüter et al. (2023), and will only briefly be summarized here: The bacteria were grown to an OD_600_ of 0.5 and resuspended in transformation/co-culture medium (TCM: ½ MS salts (NaFeEDTA 18.35 mg L^−1^), glucose (D + monohydrate) 15 g L^−1^, vitamins (myo-inositol 100 mg L^−1^, glycine 2 mg L^−1^, nicotinic acid 0.5 mg L^−1^, thiamine HCl 0.1 mg L^−1^, pyridoxine HCl 0.5 mg L^−1^), pH 5.8) with 39 mg L^−1^ acetosyringone (Roth, Germany) to an OD_600_ of 0.5 ± 0.01. The shoots were subcultured 14 days before transformation and leaf explants were cut and stored in 100 mL TCM for a maximum of 6 h, until they were used for transformation. Leaf explants and bacteria were sonicated together for 1 min in an ultrasonic bath (Bandelin Sonorex Super 10 P, type DK 102 P, Germany) with 35 kHz and 30 W L^−1^. After incubation of the explants together with the bacteria for 30 min at room temperature, leaves were placed with the adaxial side down onto solid TCM (15 leaves per vessel) (Plant Agar 7.5 g L^−1^, acetosyringone 39 mg L^−1^) and incubated for 3 d at 25 °C in the dark. Afterward, they were transferred with the same orientation onto root induction medium (10 leaves per vessel), which consisted of ½ MS salts (NaFeEDTA 18.35 mg L^−1^), sucrose 20 g L^−1^, vitamins (myo-inositol 100 mg L^−1^, glycine 2 mg L^−1^, nicotinic acid 0.5 mg L^−1^, thiamine HCl 0.1 mg L^−1^, pyridoxine HCl 0.5 mg L^−1^), indole-3-butyric acid 0.1 mg L^−1^, pH 5.8, plant agar 7.5 g L^−1^, cefotaxime 200 mg L^−1^, Timentin 100 mg L^−1^ and kept at 25 °C in the dark for 4 weeks.

### Phenotypic data analysis and visualization

Four weeks after co-culture, several traits were quantified. These included data of total and fluorescent callus (yes/no) and root formation (number), assessed separately for both laminae and petioles. The collected phenotypic data can be accessed as supplementary material in Figshare (supplementary_data.xlsx) via 10.6084/m9.figshare.25559610. For all phenotypic data of laminae and petioles, six traits were subsequently analyzed: the percentage of explants forming callus (total and fluorescent), the percentage of explants forming roots (total and fluorescent), and the number of roots per rooted explant (total and fluorescent). For the analysis of the latter, only explants with at least one root and only genotypes with at least two rooted explants were included. This was done to ensure the reliability of the data without losing too many data points.

All analyses were performed with the software R (version 4.3.0) and the interface RStudio (version 2023.12.1). Figure generation was facilitated by the use of ggplot2 [version 3.4.4, Wickham (2016)] and patchwork [version 1.2.0, Pedersen (2023)]. For correlation analysis of HR formation data, Pearson’s correlation coefficients were calculated using the R package PerformanceAnalytics [version 2.0.4, Peterson et al. (2020)]. This analysis was performed between measurements of fluorescent and total events across both organ types and all three traits. For the correlation analysis of total HR formation with AR formation of all 104 genotypes, data from Wamhoff et al. (2023b) were used. These data were derived from in vivo rooting experiments using cuttings of the same genotypes, where rooting percentages, numbers, and fresh masses were reported.

The Variance Component Analysis was carried out using the R package VCA [version 1.5.1, Schützenmeister and Piepho (2012)]. Binomial traits (percentages) were fitted using the remlMM function, with genotype, panel, and experiment included as fixed effects. For root number analysis, vessel was added as fourth effect to the model. Variance components were then calculated with the function VCAinference, using “satterthwaite” as input for ci.method. To identify the significance of the investigated factors, an Analysis of Deviance was calculated. First, we fitted generalized linear mixed models with the lme4 R package (version 1.1–33; Bates et al. 2015). The fixed effects experiment, genotype and panel were analyzed with separate models, using genotype as random effect for the experiment model and experiment as a random effect for the genotype and panel model. This approach was chosen, since a model that would include all factors would have been computationally too expensive. For binomial callus or root formation data, a binomial distribution was assumed, whereas root number per rooted explant was analyzed under the assumption of a negative binomial distribution with vessel as an additional random effect. After model fitting, an Analysis of Deviance was performed using the ANOVA function (type II) of the R package car (version 3.1–2; Fox and Weisberg 2019).

### Marker–trait association analyses

The 68 K WagRhSNP Axiom array (Koning-Boucoiran et al. 2015) was used to analyze SNPs (single-nucleotide polymorphisms). The R packages SNPpolisher [version 1.5.2, Nicolazzi et al. (2014)] and fitTetra [version 1.0, Voorrips et al. (2011)] were used to call tetraploid allele dosages. This dataset is available as supplementary material in Figshare (Called_allele_dosages.txt). The allele dosage values were further processed and filtered to ensure high data quality. For each SNP, two separate markers were used to target the SNP on both DNA strands. Each of these markers provided an allele dosage value. This was unified using the direct value if both markers provided the same value, using the value of one marker if the other was missing, or marking it as "NA" for later filtering or imputation if both markers provided different values. SNPs with minor allele frequencies below 5% and missing call rates above 10% were filtered out. In addition, no genotype exceeded a missing call rate of 20%. This resulted in a total number of 23,419 SNPs, with no monomorphic SNPs present. Missing values (NA) were imputed using the R package missForest [version 1.5, Stekhoven and Bühlmann (2012)]. The inputs used for the missForest function were maxiter = 10 and ntree = 100.

The filtered SNP data were used for GWAS analyses, which were performed using the R package GWASpoly [version 2.13, Rosyara et al. (2016)]. In these analyses, phenotypic data consisted of estimated means for each genotype. For percentage data, generalized linear mixed-effects models were fitted with "genotype" as a fixed effect and the respective transformation experiment number as a random effect, using a binomial distribution. For root number data, generalized linear mixed models were fitted using the R package glmmTMB [version 1.1.7, Brooks et al. (2017)] with “genotype” as a fixed effect and each transformation experiment number as a random effect, using the distribution “truncated_nbinom1”. After fitting each model, means were estimated using the function estimate_means from the R package model-based [version 0.8.6, Makowski et al. (2020)]. The kinship leave-one-chromosome-out (K.loco) method, one principal component, and the market segment (garden or cut rose) as fixed effects were implemented as covariates in GWASpoly to account for population structure. Quantile–quantile (QQ) plots were used to assess the chosen effects for the GWAS models. These plots showed that the majority of the *p* values followed the expected distribution for random data, indicating no major inflation. A significance level of *p* < 0.05 was applied using the M.eff method in GWASpoly, considering linkage disequilibrium (LD) together with the Bonferroni adjustment. Association mapping was performed assuming an additive allele dose–trait relationship, since dominant relationships showed poorer results in QQ plots.

### Peak selection and analysis

In addition to significantly associated SNP markers, genomic regions of interest that exhibited non-significant but distinctive peaks were analyzed. Peaks were selected, when *p* values from SNP associations reached 80% of the significance threshold. The region of interest for SNP analysis was restricted to the full width at half maximum of each peak. The identified peaks were subjected to detailed analyses. The contribution of each peak to the variability of the phenotype was determined using the fit.QTL function from the R package GWASpoly, using the SNP with the strongest association per peak. Direct effects on the respective traits were also estimated, following the methodology of Wamhoff et al. (2023b). This involved several steps: only SNPs with at least four out of five possible allele dosage groups (ADGs) and at least five genotypes per ADG were considered. Selected SNPs were tested for significant differences depending on the allele configuration using the Kruskal–Wallis test (*p* > 0.05). SNPs found to be significant were further analyzed using Fisher's Least Significant Difference (LSD) criterion with the function LSD.test from the R package agricolae [version 1.3–5, De Mendiburu (2023)]. This was done to determine which ADGs were significantly different from each other (*p* > 0.05, Holm–Bonferroni adjustment). Trait effect sizes were calculated between the most different ADGs for selected SNPs that showed significant differences for the traits of interest.

### Candidate gene identification

For the identification of CGs, 341 published genes with known effects on transformation or *Rhizobium* infection were blasted against the rose genome (Crane and Gelvin 2007; Karami et al. 2009; Soltani et al. 2009; Pitzschke 2013; Bourras et al. 2015; Willig et al. 2018; Gelvin 2021). The gene names are listed in the supplementary material in Figshare (supplementary_data.xlsx). First, gene sequences were obtained from the NCBI gene database using the R package rentrez (version 1.2.3, Winter (2017)) with the search term “(gene_name[Gene Name] OR gene_name[Gene Description] OR gene_name[sym]) AND Spermatophyta[ORGANISM]". If there were multiple hits, the gene ID from *Arabidopsis thaliana* was used, or if *Arabidopsis thaliana* was not present, the first hit was used. Gene names for which no gene database ID was found were manually checked with other search terms or with help of the TAIR database, to find a corresponding NCBI gene ID. Duplicate gene IDs were removed and DNA sequences were retrieved, also using the R package rentrez. As reference sequences for the BLAST, rose gene sequences were obtained from the “Genome database for Rosaceae” GDR (https://www.rosaceae.org/species/rosa/chinensis/genome_v1.0, last accessed 7th of April, 2024). Gene sequences were blasted against the selected peak regions of the rose genome using the R package metablastr [version 0.3.1, Benoit and Drost (2021)] with the function blast_best_hit, and the results were filtered for qcovhsp ≥ 50, to ensure a sufficient BLAST hit quality.

The identification of CGs was also approached using differentially expressed gene (DEG) datasets from the Gene Expression Omnibus database (https://www.ncbi.nlm.nih.gov/gds, last accessed 30th of June, 2024) with the query “hairy roots OR ((agrobacterium OR rhizobium) AND (transformation OR infection))”. Results were filtered for “series”, in following renamed as “studies”, with suitable study design and accessibility of DNA sequences of the probes or underlying genes, either from submitted files or platform (chip) data. In cases where only *Arabidopsis* identifiers were provided, DNA sequences were obtained using the R package rentrez as previously described. For studies with the option GEO2R (GSE32426, GSE14106, GSE4116, GSE62751), samples were combined according to their respective groups, and contrasts for the calculation of the differential expression were selected as listed in the supplementary data file. *P* value calculations were performed based on default GEO2R settings and datapoints with *p* < 0.05 were downloaded from the GEO2R volcano plot option. Significant DEG datapoints from studies without GEO2R option were extracted from the supplementary materials [GSE179628, Liu et al. (2021), “Agrobacteria-responsive genes”; GSE172314, Lapham et al. (2021), “Supplemental Data Sheet 1.XLSX”—sheet “VirE2 Up with Agro” and “VirE2 Down with Agro”]. Ex- and inclusion criteria and GEO2R contrasts for each study as well as links to the sequence retrieval files are listed in the supplementary material in Figshare (supplementary_data.xlsx). DEG chip spot identifiers were combined with their respective DNA sequences. BLAST analysis of the DEG sequences against the peak regions was conducted as previously explained. Potential CGs were defined as genes in rose genome peaks that could be blasted against DEGs in at least two different transcriptomic studies.

To identify corresponding *Arabidopsis* genes and the respective gene ontology (GO) biological processes, CG coding sequences were blasted against the Araport11 coding sequences (DNA) database (https://v2.arabidopsis.org/Blast/, last accessed 01st of August, 2024). The top hit was taken to extract the *Arabidopsis* ID and the GO biological process.

## Results

### Root and callus formation

The optimization of the transformation protocol and the experimental set-up were previously reported by Rüter et al. (2023). In brief, 104 rose genotypes from two different panels, cut roses and garden roses, were transformed with the *R. rhizogenes* strain ATCC 15834 harboring a binary vector carrying the reporter gene E*GFP*. This gene served as an indicator of successful HR formation.

**Fig. 1:**
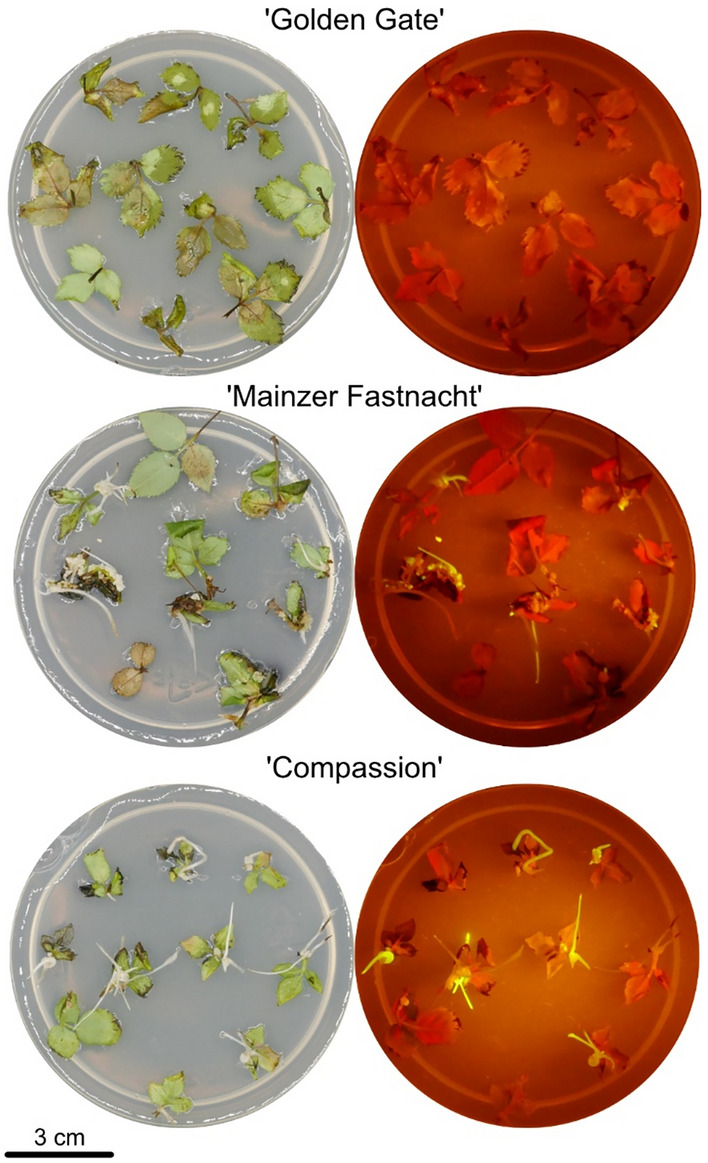
Genotypes differing in hairy root formation capacity. Shown are top view photos of leaf explants, four weeks after co-culture with *R. rhizogenes* strain ATCC 15834:GFP. Next to each bright light photo (left column) there is a photo of blue-green-LED excited fluorescence of hairy roots expressing GFP photographed with an orange filter (right column) (colour figure online)

Several experimental factors were investigated for hairy root formation: rose explant types, wounding via sonication, different *R. rhizogenes* strains, and the effect of Silwet L-77. This optimized protocol was then used for hairy root induction in 104 genotypes, revealing a strong genotypic effect on compound leaf explants (Rüter et al. 2023). The present study examined hairy root formation in greater detail by separately analyzing laminae and petioles. Additionally, a GWAS was performed to identify QTLs affecting these traits. Figure 1 shows visual examples of genotypes with divergent capacities for HR formation.

**Fig. 2:**
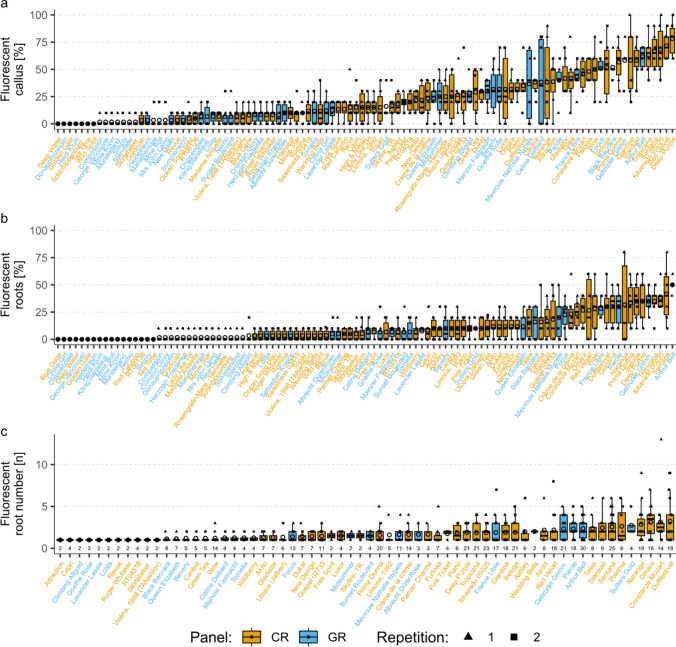
Phenotypic data for fluorescent callus and root formation from laminae of leaf explants of different rose genotypes. Circles as means, CR and GR representing the cut rose or garden rose panel. 104 genotypes for callus percentage data, 104 genotypes for root percentage data, 64 genotypes for data of root numbers per rooted explant, where only genotypes with at least two rooted explants were considered. N for percentage data = 3 vessels with 10 explants in each of two repetitions. N for root counting data is mentioned for each genotype below the boxplot

**Fig. 3:**
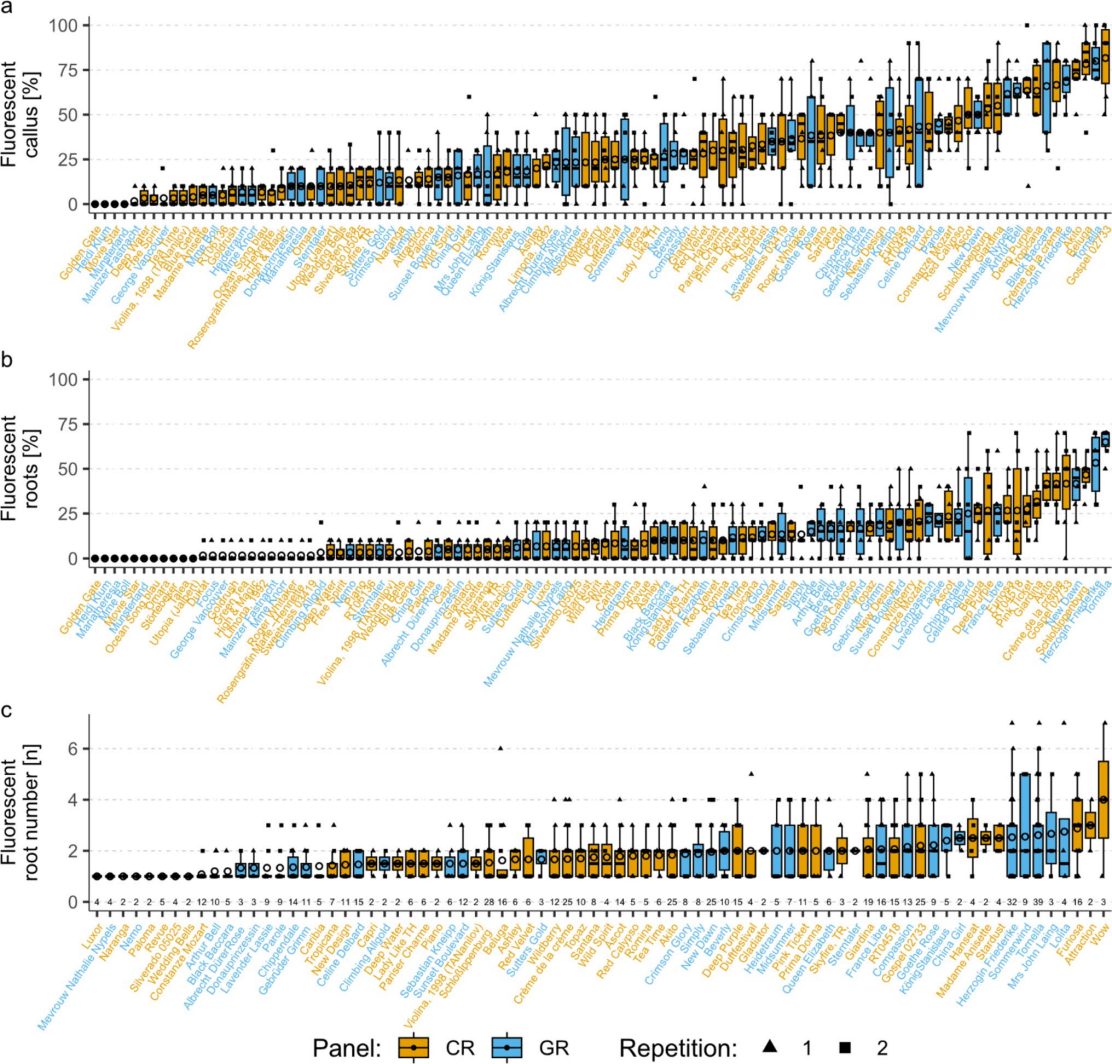
Phenotypic data for fluorescent callus and root formation from petioles of leaf explants of different rose genotypes. Circles as means, CR and GR representing the cut rose or garden rose panel. 104 genotypes for callus percentage data, 104 genotypes for root percentage data, 78 genotypes for data of root numbers per rooted explant, where only genotypes with at least two rooted explants were considered. N = 3 vessels with 10 explants in each of two repetitions. N for root counting data is mentioned for each genotype below the boxplot

Phenotypic data for the association study were provided by measurements of total events (Figs. S1 and S2) and events expressing fluorescence (Figs. 2 and 3), with regard to callus (CF%) and root (RF%) formation, and the number of roots per rooted explant (RN) of laminae and petioles. Strong genotypic differences were observed for all measured parameters, both for total and fluorescent data. However, since data on only fluorescent callus and roots indicated proven transformation events, this data is referred to in the subsequent analysis.

In the case of laminae, CF% was observed in 0–78.3% of the explants (mean ± SD (standard deviation): 22.0 ± 19.9%), with the cut rose genotype ‘Deep Purple’ showing the highest percentage (Fig. 2a). RF% varied from 0 to 50% (mean: 10.1 ± 11.6% SD), with the garden rose ‘Arthur Bell’ at the upper end of this range (Fig. 2b). Up to 3.2 RNs were counted (mean: 1.7 ± 0.6 SD), with the cut rose ‘Duftfestival’ having the highest count (Fig. 2c). When considering petioles, the cut rose ‘Gospel 02733’ exhibited the highest CF%, whereas the whole set ranged from 0 to 81.7% (mean: 27.2 ± 20.3% SD) (Fig. 3a). The garden rose ‘Tornella’ had the highest value of RF% on petioles, while the total range was 0–65% (mean: 11.9 ± 12.7% SD) (Fig. 3b). The cut rose ‘WOW’ had the highest RN with a value of 4 (mean: 1.8 ± 0.6 SD) (Fig. 3c).

The substantial effect of the genotype on the total variance was confirmed by a variance component analysis. This analysis revealed a stronger influence of the genotype compared to panel, experiment, or vessel, especially for CF% and RF% (Table S1). Unknown factors (error) were also found to have a significant effect on phenotypic variability, especially for the RN (84–92%). To determine the significance of the factors genotype, rose panel, and experiment on all traits, an analysis of deviance was performed. The results were largely consistent with the results of the variance component analysis, showing a significant effect of the genotype on all traits except for the fluorescent RN of petioles [*p* = 0.33 (Table S2)]. Interestingly, significant effects of the factors panel and experiment were observed for some traits, although these effects were judged to be small based on the variance component analysis. In several cases, the distribution of cut and garden rose genotypes was not homogeneous for the measured traits. This was evident when comparing *p* values for fluorescent CF% and RF%, where the panel appeared to have a significant effect on laminae but not on petioles.

### Correlations between traits and with adventitious root formation

Correlations between measurements of fluorescent and total regeneration events across both organ types and the three traits were analyzed (Fig. S3). A high correlation between fluorescent and total roots would indicate a stable portion of HR among all roots. Strong correlations were observed between fluorescent and total CF% and RF%. Correlation values ranged from 0.85 to 0.9 for three parameters: CF% on lamina, RF% on lamina and RF% on petiole. Medium correlations (ranging from 0.48 to 0.62) were found for CF% on petiole and RN on both lamina and petiole. When comparing specifically between lamina and petiole organs, only a weak correlation (0.42) was observed for CF%. No correlation was found between the two organ types for rooting parameters.

All genotypes had previously been submitted to GWASs by Wamhoff et al. (2023a), Nguyen et al. (2020b) and Nguyen et al. (2020a). These analyses focused on the ability of genotypes to form roots on cuttings and roots or callus on shoots in tissue culture. In all studies, including this one, strong genotypic variation was consistently reported for all observed traits. Thus, also correlations between HR formation data and AR formation data from Wamhoff et al. (2023b) were examined. In their study, in vivo cuttings of the same genotypes were rooted and root formation percentages, numbers and fresh masses were reported. Correlations of AR with HR data were present but weak, as shown in Fig. S4. Only a negative correlation of − 0.2 was observed for RN of laminae with the RN of cuttings. The traits recorded for petioles showed stronger correlations with their corresponding in vivo traits. A correlation coefficient of 0.3 was found between the RF% of petioles and RF% of cuttings. Similarly, a correlation coefficient of 0.32 was observed between the RN of petioles and RN of cuttings.

### Marker–trait association analyses

**Fig. 4:**
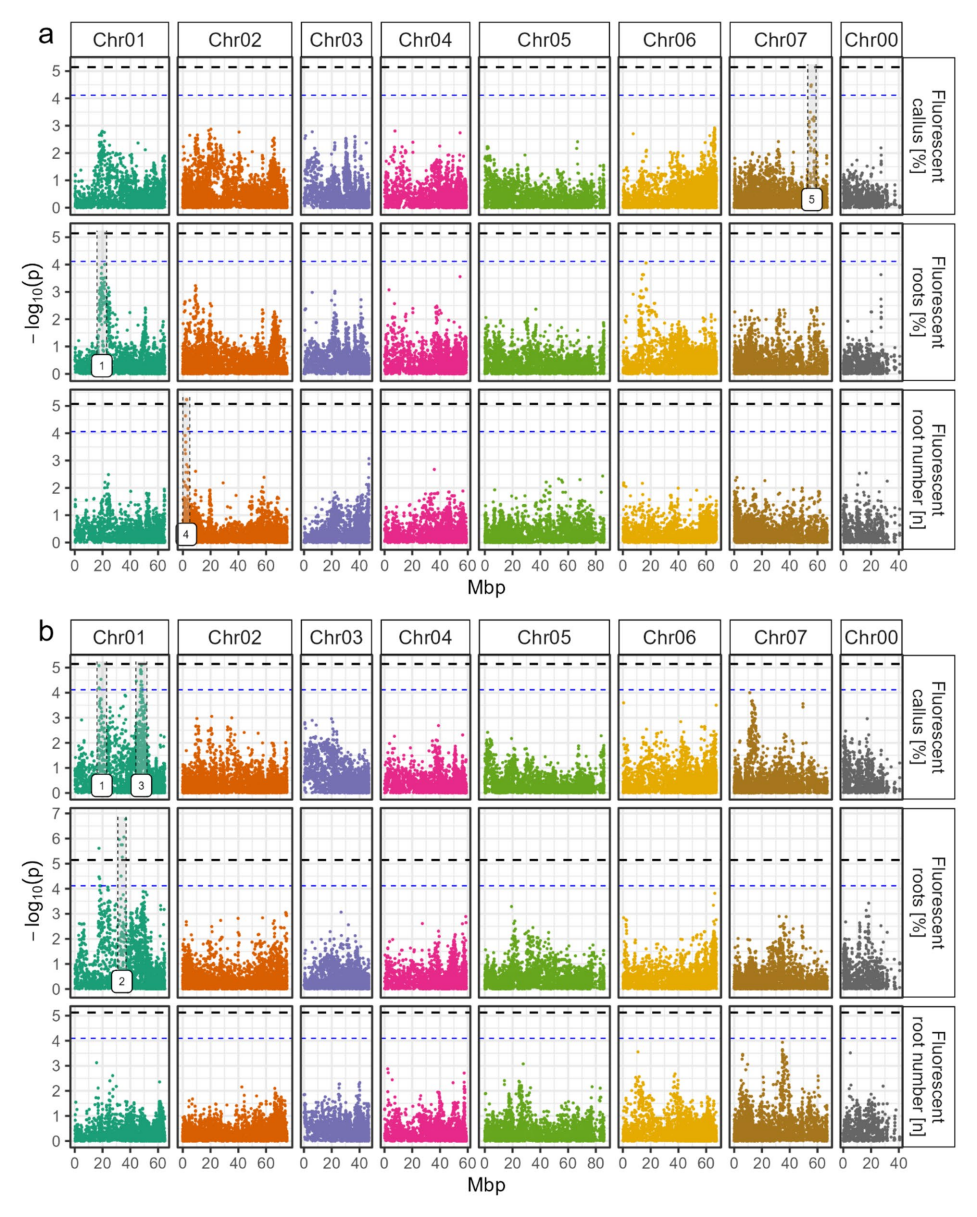
Manhattan plots with the results of marker and fluorescent trait associations. 23,419 SNPs were used for the association analysis, utilizing an additive model and depicted as − log10 of the SNP´s specific *p* value. **a** Data from laminae. **b** Data from petioles. The x-axis shows the positions with respect to the seven *Rosa chinensis* chromosomes (Hibrand Saint-Oyant et al. 2018) (Chr01-Chr07) in megabase pairs (Mbp). Chr00 covers contigs with SNPs that have not yet been mapped. Root numbers were taken only from root forming explants and genotypes with at least two rooted explants. The horizontal dashed black line indicates the M.eff corrected *p* value significance threshold, the blue line indicates 80% of the significance threshold for peak selection. Selected peaks are highlighted with gray backgrounds and numbered from left to right (colour figure online)

A marker–trait association analysis was conducted, in which the phenotypic data of the fluorescent events were associated with the marker data. These GWASs were performed separately for all traits and for the two different organs, lamina and petiole, as shown in Fig. 4a and b, respectively. The associations of the data of the total events were also examined and visualized in Fig. S5. The analyses did not reveal a single dominant peak associated with any trait for any organ. Instead, several distinct peaks were identified, some of which reached the significance threshold. This suggests the investigated traits have a complex genetic architecture. Significant SNPs were discovered in the peak on chromosome two for the RN of laminae (Fig. 4a) and on chromosome one for the RF% of petioles (Fig. 4b). All significant SNPs are listed in Table 1. Intriguingly, some peaks were found in the same genomic region for several traits. For example, on chromosome one, the regions around 19 Mbp and 51 Mbp exhibited some degree of association with most traits in both organ types. Particularly for the CF% of petioles (Fig. 4b), the analyses identified prominent peaks that showed strong associations, nearly reaching the significance threshold. This suggests that these genomic regions may play a role in callus formation. When comparing petiole and lamina, hardly any shared peaks were observed with the exception of the aforementioned peaks on chromosome one (Fig. 4a versus b). This observation supported the outcomes of the correlation analysis.

**Table 1:** Significant SNPs located in distinct peaks in Manhattan plots in Fig. 4 with their respective gene predictions

Marker	Peak	Chr	Position	− log_10_ (*p*)	Trait	Gene prediction
Rh12GR_24085_427	1	1	17454693	5.61	RF% P	Myb domain protein 33
Rh12GR_83541_164	2	1	32001537	5.96	RF% P	Fatty acid hydroxylase superfamily
RhK5_507_2124	2	1	34254886	5.75	RF% P	RNA-binding (RRM/RBD/RNP motifs) family protein
RhK5_507_1881	2	1	34255631	5.26	RF% P	RNA-binding (RRM/RBD/RNP motifs) family protein
RhK5_444_1479	2	1	35399153	6.06	RF% P	Transcription factor jumonji (jmjC) domain-containing protein
RhK5_3761_1470	2	1	33400030	5.75	RF% P	Metacaspase-1
RhMCRND_17105_1110	2	1	36597801	6.77	RF% P	Unknown—blast not successful
RhK5_6546_859	4	2	2711391	5.23	RN L	Plant-specific transcription factor YABBY family protein
RhK5_684_1781	4	2	2800338	5.23	RN L	EIN3-binding F box protein 1
RhK5_684_2502	4	2	2799617	5.23	RN L	EIN3-binding F box protein 1

Corresponding gene predictions were obtained from the “Genome database for Rosaceae” GDR (https://www.rosaceae.org/species/rosa/chinensis/genome_v1.0, last accessed 7th of April, 2024). Genes without prediction were blasted via NCBI. RF%, percentage of root formation; RN, root number of rooted explants; P, petiole; L, lamina

### QTL influence on the phenotype

The number and comparable height of all peaks observed in the Manhattan plots suggested, that the measured traits were not influenced by a single genomic region, but rather by numerous QTLs. Based on these findings, a subset of the most important peaks was selected for further analysis, which were numbered from one to five based on their location in the genome and are shown in the Manhattan plots in Fig. 4. These selected peaks exhibited several interesting characteristics. For example, peaks one and three were strongly associated with fluorescent CF% on petioles (Fig. 4b), but not with total CF% (Fig. S5b). Peak four was detected for the fluorescent RN of laminae (Fig. 4a), but not for the total RN (Fig. S5a). Additionally, this peak was observed in the data collected from laminae, but not from petioles. Peak five was present in data from laminae but not petioles and was present in both, fluorescent (Fig. 4a) and total (Fig. S5a) CF% of laminae.

The contribution of each peak to the phenotypic variability of the different traits was also estimated. The analysis was facilitated by the R package GWASpoly, which allowed the evaluation of the influence of the strongest SNP within each peak on the variability of the traits. Upon examination, all peaks showed a significant influence on the variability of the respective trait. This influence ranged from 12.0% (peak one, fluorescent RF% of laminae) to 26.9% (peak four, fluorescent RN of laminae) (Table S3).

**Fig. 5:**
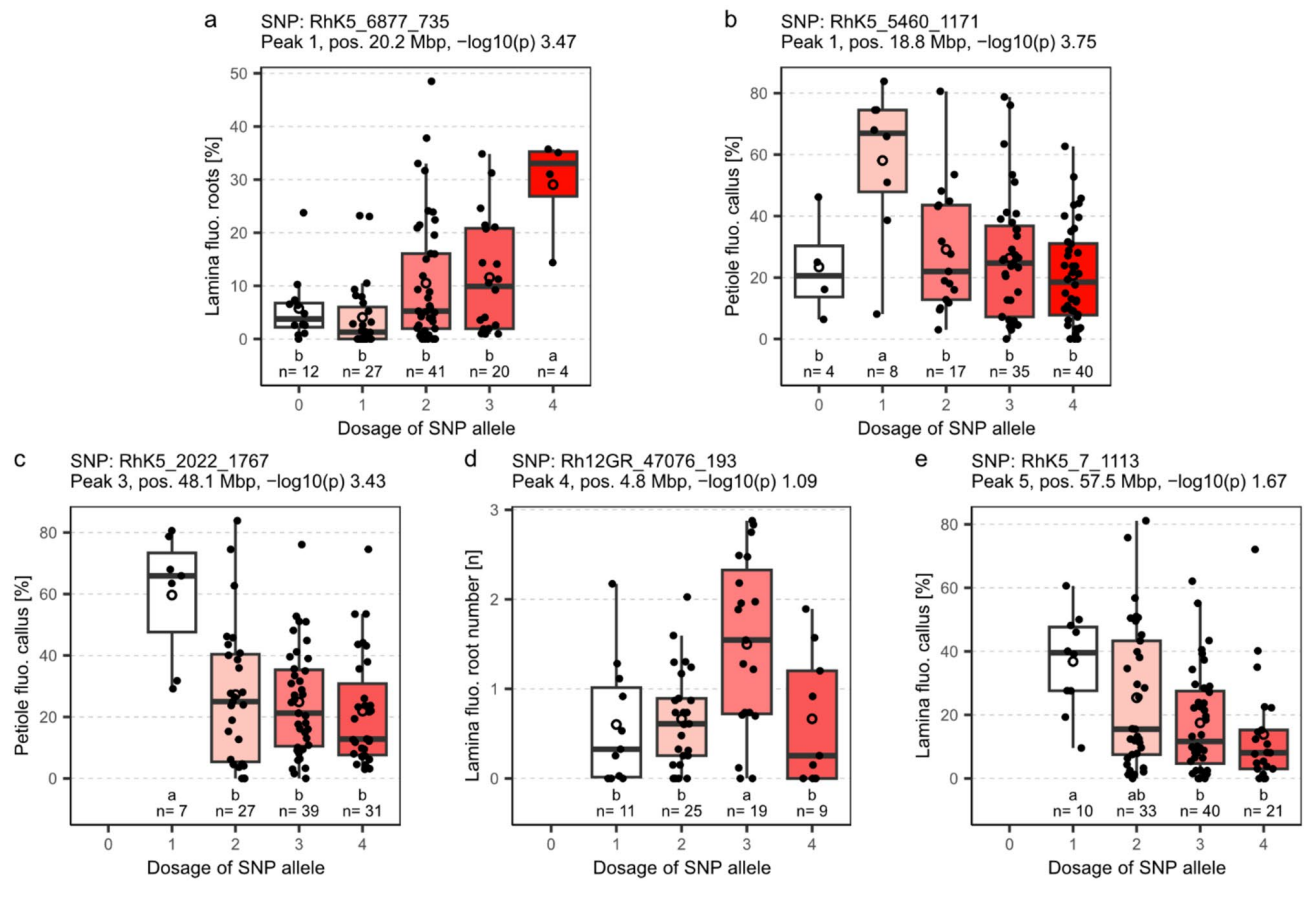
Effects of allele dosage configuration for SNPs with the strongest significant effects for each peak and trait. **a** Peak 1 for root formation percentage on laminae. **b** Peak 1 for callus formation percentage on petioles. **c** Peak 3 for callus formation percentage on petioles. **d** Peak 4 for root numbers of rooted explants on laminae. **e** Peak 5 for callus formation percentage on laminae. For peak two, no significant effects were found. X-axis values show the dosages for the SNP allele from nulliplex (0) to quadruplex (4). The number of individuals per allele dosage group is given by n. Letters indicate significance groups as determined by Fisher’s LSD criterion for *p* < 0.05 under consideration of the Holm–Bonferroni adjustment separately for each trait

In addition to the peak contribution on total variability, potential effects on the traits were estimated. Since a limited number of genotypes were analyzed, ranging from 64 to 104, this resulted in some SNPs not being represented in all ADGs. The SNPs with the strongest effects for each peak are shown in Fig. 5 and listed in Table S4, along with the respective underlying gene predictions. The exception is peak two, for which no SNP was identified that showed significant differences between ADGs. Each of these SNPs showed a significant influence of the allele dosage configuration on the respective trait. In particular, SNP RhK5_6877_735 in peak one showed a clear trend with each additional allelic exchange from ADG zero toward ADG four (Fig. 5a), with a difference in means of 25% fluorescent RF% on laminae between ADG one and four. The strongest effect was shown by SNP RhK5_2022_1767 in peak three (Fig. 5c), with a difference in means of 38% fluorescent CF% on petioles between ADG one and four.

### Candidate gene identification

While SNP markers are sufficient for breeding purposes, a deeper understanding of the transformation and HR formation process requires the identification of CGs. This identification can allow the design of a follow-up functional study to test and validate these genes. A common approach for CG identification is the evaluation of genes, in which significantly associated SNPs are present. All ten significant SNPs with their underlying gene predictions are listed in Table 1.

Two additional approaches for CG identification were followed as well. First, 341 published genes with an hypothesized or proven impact on transformation or *Rhizobium* infection (Crane and Gelvin 2007; Karami et al. 2009; Soltani et al. 2009; Pitzschke 2013; Bourras et al. 2015; Willig et al. 2018; Gelvin 2021) were blasted against the peak regions in the rose genome. The second approach involved blasting DEGs from published transcriptomic datasets with experimental designs suitable for investigation of *Rhizobium* transformation or infection against the peak regions (Gene Expression Omnibus series: GSE179628, GSE172314, GSE32426, GSE14106, GSE4116, GSE62751). Ten interesting CGs were found in the aforementioned peaks, which are listed together with their respective GO biological processes in Table 2.

**Table 2:** Candidate genes (CGs) identified in peaks by BLAST

Peak	Gene predictions	Rose ID	CG reference	*Arabidopsis* ID	GO biological process
2	*Heat shock protein 70*	RC1G0246600	GSE62751, GSE179628	AT3G12580	Protein folding, response to bacterium, response to heat, response to temperature stimulus, response to virus
3	*Calcium-binding EF-hand family protein*	RC1G0386900	GSE14106, GSE62751, GSE172314	AT2G46600	Trichome branching
	*3-Ketoacyl-CoA synthase 1*	RC1G0390900	Willig et al. (2018)	AT1G01120	Cellular response to cold, cutin-based cuticle development, fatty acid elongation, response to cold, response to light stimulus, very long-chain fatty acid biosynthetic process, very long-chain fatty acid metabolic process, wax biosynthetic process
	*Acyl-CoA N-acyltransferases (NAT) superfamily protein*	RC1G0411600	GSE14106, GSE32426, GSE179628	AT2G39030	Ornithine metabolic process, response to jasmonic acid
	*Phenylalanine ammonia-lyase 4*	RC1G0412100	GSE14106, GSE32426, GSE62751, GSE179628	AT3G10340	Cinnamic acid biosynthetic process
	*Alpha/beta-hydrolases superfamily protein*	RC1G0415200	GSE14106, GSE32426	AT5G19290	Not mentioned
4	*SOS3-interacting protein 3*	RC2G0014700	GSE14106, GSE62751	AT4G30960	Basipetal auxin transport, hyperosmotic salinity response, multicellular organism development, response to salt stress, response to water deprivation
	*Raffinose synthase family protein*	RC2G0031500	GSE62751, GSE179628	AT5G20250	Cellular response to hypoxia, response to cold, response to oxidative stress
	*Nucleotide-sugar transporter family protein*	RC2G0039600	GSE32426, GSE62751	AT4G32390	Transmembrane transport
	*Receptor-like kinase*	RC2G0044800	Willig et al. (2018)	AT5G20050	Phosphorylation

Used were published genes with a putative impact on transformation processes or genes reported in at least two transcriptomic datasets which were differentially up- or downregulated during *Rhizobium* transformation or infection. Gene predictions and rose gene IDs were taken from the “Genome database for Rosaceae” GDR (https://www.rosaceae.org/species/rosa/chinensis/genome_v1.0, last accessed 7th of April, 2024)

## Discussion

All the traits analyzed in this study were shown to be strongly influenced by the genetic background of the 104 rose genotypes. This was true for both laminae and petioles, and for both total and fluorescent data. The traits measured appear to have a complex genetic basis caused by multiple loci. This complexity is evidenced by the continuous variation of each trait observed in the population studied. Likewise in the Manhattan plots, no major peak was detected, but rather several smaller peaks with similar association strengths were found. Peaks one and three on chromosome one appeared in most traits linked to HR formation. This hints at common QTLs, though with small effect, which probably affect HR formation at a fundamental level. Furthermore, these two peaks were the only ones that showed remarkable association for both organ types—lamina and petiole. The other peaks did not show this pattern, as lamina and petiole generally did not share common peaks.

### Similarities to AR formation

The aforementioned peaks one and three on chromosome one were also reported to play a role for the number of ARs in a garden rose panel in an in vitro system (Nguyen et al. 2020a) and for AR formation percentages in cuttings of cut and garden roses in an in vivo hydroponic system (Wamhoff et al. 2023b). Peak four on chromosome two was likewise reported in both studies to play a role for root number and root biomass (Nguyen et al. 2020a) as well as for root formation (Wamhoff et al. 2023b). The remaining peaks, peak two on chromosome one at around 32 Mbp and peak five on chromosome seven around 55 Mbp, were not found in any of the GWAS reports for root or callus formation on cut and garden rose genotypes. Furthermore, no QTL from the callus study of Nguyen et al. (2020b) was found to be involved during root or callus formation in this study, which might be an indication for QTLs with a genetic influence on the transformation process.

QTL linkage studies conducted on rapeseed using *A. tumefaciens* and *R. rhizogenes* confirmed that HR formation is a genetically complex trait influenced by multiple QTLs, each with small effects (Cogan et al. 2002, 2004; Oldacres et al. 2005). These studies identified QTLs responsible for the process of AR and HR formation, explaining up to 26% of the total variation. It was also observed that genotypes predisposed toward AR formation also demonstrated a high prevalence of HR formation. Similar results were observed in the present study. The peaks contributed 12.0%–26.9% to the total variability, depending on the respective trait. In addition, positive correlations were found between HR formation on petioles and AR formation on cuttings. However, the rather weak to moderate correlation of HR and AR formation can be explained with the genetic complexity of both traits, as only few of the responsible loci are associated with both traits. Moreover, the correlations were calculated between HR formation in vitro and AR formation in vivo, i.e., in two very different trophic conditions with in vitro shoots and two-node cuttings. Likewise, Nguyen et al. (2020a) and Wamhoff et al. (2024) found only weak correlations for AR formation in vitro and in vivo.

Remarkably, no prominent peak was overlapping lamina and petiole for any trait. This profound genetic difference was mirrored in the correlation analysis, where no correlation was found for lamina and petiole traits. Additionally, correlation of HR formation on petioles and AR formation on shoot stems was observed, which was missing for laminae. Literature suggests some similarities between stems and petioles, as AR formation had been known to occur on wounded petioles, similar to stems (Correa et al. 2012). Furthermore, petioles had been reported to elongate in a similar way as stems with dark treatments (Thomas and Raper 1985; Kozuka et al. 2005), and nutrient concentration in the petiole of grapevines had been found to be more closely correlated to the stem than to the blade (Baby et al. 2021). Different factors influencing HR induction in both organ types, indicate that different genes may play crucial roles during HR formation in lamina and petiole. QTLs known for adventitious rooting might have a stronger impact on HR formation on petioles, and conversely, QTLs found in the analysis of laminae might be more related to the transformation process than to root formation.

### Candidate gene identification

One approach to identify CGs used in several GWASs (Francisco et al. 2016; Wamhoff et al. 2023b; Yu et al. 2023) is based on the genes in which significant SNPs are found. All ten significant SNPs from this study, along with their underlying gene predictions, are listed in Table 1. These genes are known for their roles in various biological processes, including plant defense mechanisms, stress responses, hormonal pathways, and developmental processes. Although these pathways sound promising, the CGs should be considered with caution, due to the limited number of genotypes and rather low saturation of SNP markers (23,419 SNPs) for a 512 Mbp genome (Hibrand Saint-Oyant et al. 2018). Several genes were not represented by SNP markers due to stringent marker filtering for the design of the SNP chip (Koning-Boucoiran et al. 2015) and the filtering steps for this GWAS.

Another approach for CG identification involved selecting genes reported in other studies with functionally validated impact on transformation or *Rhizobium* infection. These genes were subsequently blasted against the rose genome, and those present within peak regions were selected as potential CGs. A third and rather novel approach of this study to identify CGs was to examine differentially expressed genes from published transcriptomic studies. Willig et al. (2018) summarized several transcriptomic studies with focus on *Rhizobium* infection and grouped highly up- or down-regulated genes based on their gene ontologies and the type of study in which they were investigated. A similar approach was followed by blasting DEGs from published datasets of transcriptomic studies with a suitable experimental design against the peak regions. Genes were then filtered for those, which were differentially up- or downregulated in at least two studies. The identified CGs were demonstrated to play roles in diverse biological pathways, such as stress response, calcium signaling, fatty acid biosynthesis, secondary metabolite synthesis, various metabolic activities, oligosaccharide synthesis, and signal transduction. In order to highlight possible links to transformation and hairy root formation, some of their functions are listed in the following: *heat shock protein 70* is a chaperone protein crucial in plant stress response, helping in protein folding and trafficking during *Agrobacterium*-mediated transformation and plant–pathogen interactions (Usman et al. 2017). Likewise, calcium-binding proteins, including *EF-hand family proteins* (Kaur and Madhu 2022) and *SOS3-interacting protein 3* (Sánchez-Barrena et al. 2005), are integral to calcium signaling pathways in plant–microbe interactions and salt stress tolerance. The enzyme *3-Ketoacyl-CoA synthase 1* is involved in very-long-chain fatty acid biosynthesis and plant defense responses (Lee et al. 2009), being upregulated during *Arabidopsis thaliana* treatment with *Rhizobium* PAMPs and affecting cuticle composition (Willig et al. 2018), which could influence wounding treatments and bacterial attachment. These are only four of several examples where our CGs match functions related to the response of plants to *Agrobacterium* infection as listed in Table 2.

Overall, this study confirmed the complex genetic architecture underlying hairy root formation in roses following *R. rhizogenes* transformation. The association analysis identified several genomic regions that influence this trait. Some of these regions overlapped with known QTLs for adventitious rooting, consistent with the observed correlation between HR formation and AR formation. Notably, the differential responses observed between laminae and petioles suggest organ-specific genetic control mechanisms. The identified CGs predominantly relate to stress responses and early infection processes. These findings advance our understanding of the genetic basis of hairy root formation. They also highlight potential targets for improving transformation efficiency in roses and possibly other ornamental crops. Future research should focus on functional validation of these CGs and exploration of their roles both in *Rhizobium* infection and in broader plant–microbe interactions. It would be particularly valuable to investigate how these CGs interact with bacterial *rol* genes and other genes on the T-DNA, as this would significantly advance our understanding of their function. This work lays a foundation for developing strategies to improve genetic transformation techniques in roses, potentially accelerating breeding programs and facilitating more efficient production of transgenic plants for both research and commercial applications.

## Supplementary Information

Below is the link to the electronic supplementary material.

Supplementary file1 (DOCX 4503 KB)

## Data Availability

The GWAS experimental data (SNP data, genotype codes, callus and root counts) and data used for candidate gene identification (gene names from publications, information about DEG datasets) that support the findings of this study are available in the repository Figshare with the following DOI: 10.6084/m9.figshare.25559610.

## Abbreviations

ADG: Allele dosage group
AR: Adventitious root
C: Callus
CF%: Percentage of explants forming callus
CG: Candidate gene
Chr: Chromosome
CR: Cut rose
DEG: Differentially expressed gene
GDR: Genome database for Rosaceae
GFP: Green fluorescent protein
GO: Gene ontology
GR: Garden rose
GWAS: Genome-wide association study
HR: Hairy root
ID: Identifier
L: Lamina
Mbp: Mega base pairs
n: Number
P: Petiole
PAMP: Pathogen-associated molecular pattern
QTL: Quantitative trait locus
R: Root
RF%: Percentage of explants forming roots
Ri: Root inducing
RN: Number of roots per rooted explant
rol: Root oncogenic locus
SD: Standard deviation
SNP: Single-nucleotide polymorphism
T-DNA: Transfer-DNA
Ti: Tumor inducing
vir: Virulence
