# Supplementary material for: Unraveling the genetic basis of Rhizobium rhizogenes-mediated transformation and hairy root formation in rose using a genome-wide association study
Source: Plant Cell Rep. 2024 Dec 3;43(12):300. doi: 10.1007/s00299-024-03388-4 (PMC11615123; doi:10.1007/s00299-024-03388-4)
Supplement: Supplementary file 1 — Supplementary file1 (DOCX 4503 KB) [file 299_2024_3388_MOESM1_ESM.docx]

# Supplementary Information

Journal: Plant Cell Reports

Titel: Unravelling the genetic basis of *Rhizobium rhizogenes*-mediated transformation and hairy root formation in rose using a genome-wide association study

Philipp Rüter^1^, Thomas Debener^2^, Traud Winkelmann^1^

^1^Institute of Horticultural Production Systems, Section Woody Plant and Propagation Physiology, Leibniz University Hannover, Herrenhäuser Str. 2, 30419 Hannover, Germany

^2^Institute of Plant Genetics, Section Molecular Plant Breeding, Leibniz University Hannover, Herrenhäuser Str. 2, 30419 Hannover, Germany

Corresponding author: Philipp Rüter, rueter@baum.uni-hannover.de

This file contains supplementary information about phenotypic data for total callus and root formation from laminae and petioles, results of Variance Component Analysis and Analysis of Deviance to evaluate factors potentially influencing the traits, correlations of callus, hairy root and adventitious root formation data, Manhattan plots of total (non-fluorescent) data and information about relevant SNPs in peak regions.


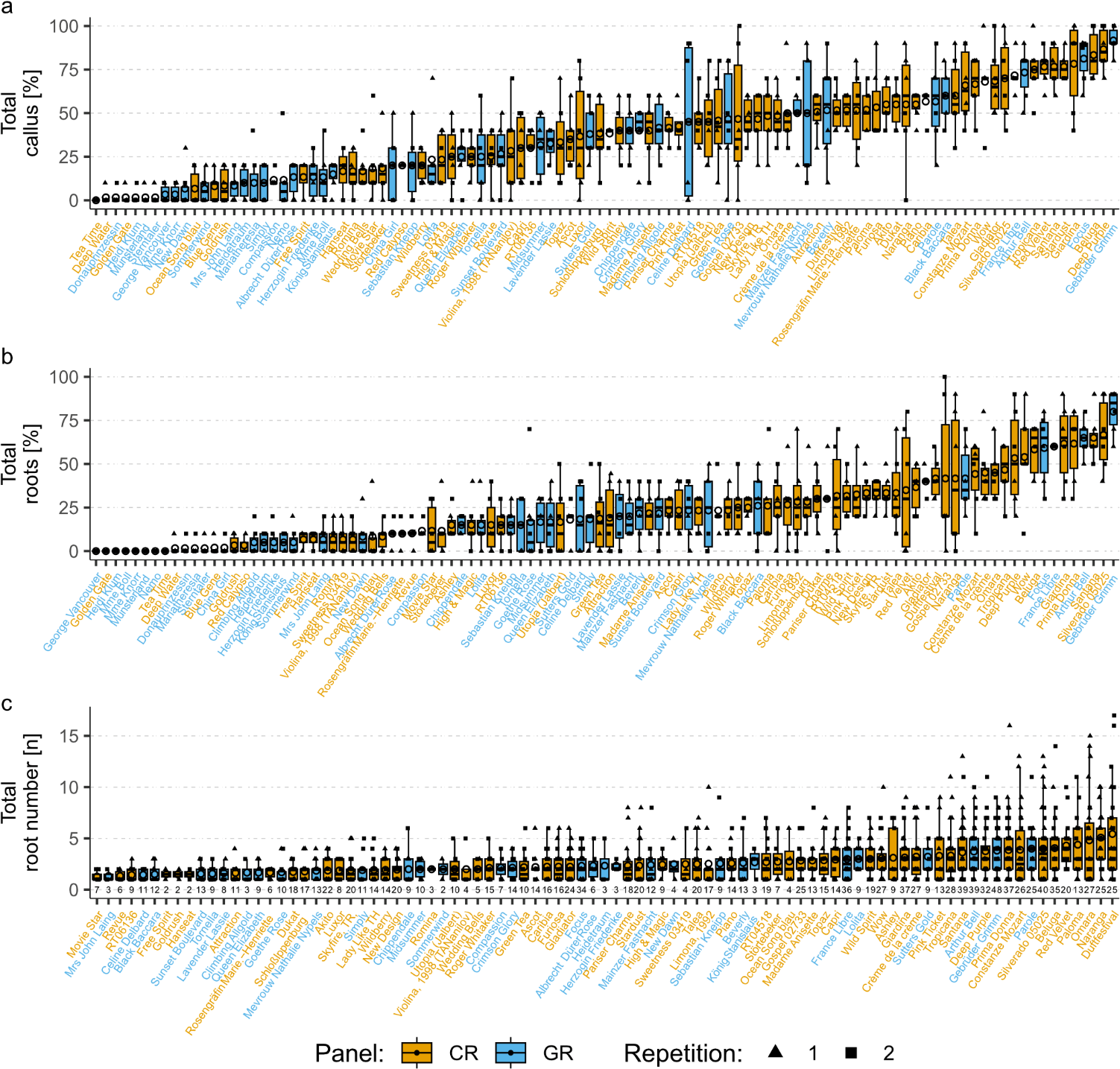


**Fig. S1** Phenotypic data for total callus and root formation from laminae of leaf explants of different rose genotypes. Circles as means, CR and GR representing the cut rose or garden rose panel. 104 genotypes for callus percentage data, 104 genotypes for root percentage data, 89 genotypes for data of root numbers per rooted explant, where only genotypes with at least two rooted explants were considered. N = 3 vessels with 10 explants in each of two repetitions. N for root counting data is mentioned for each genotype below the boxplot


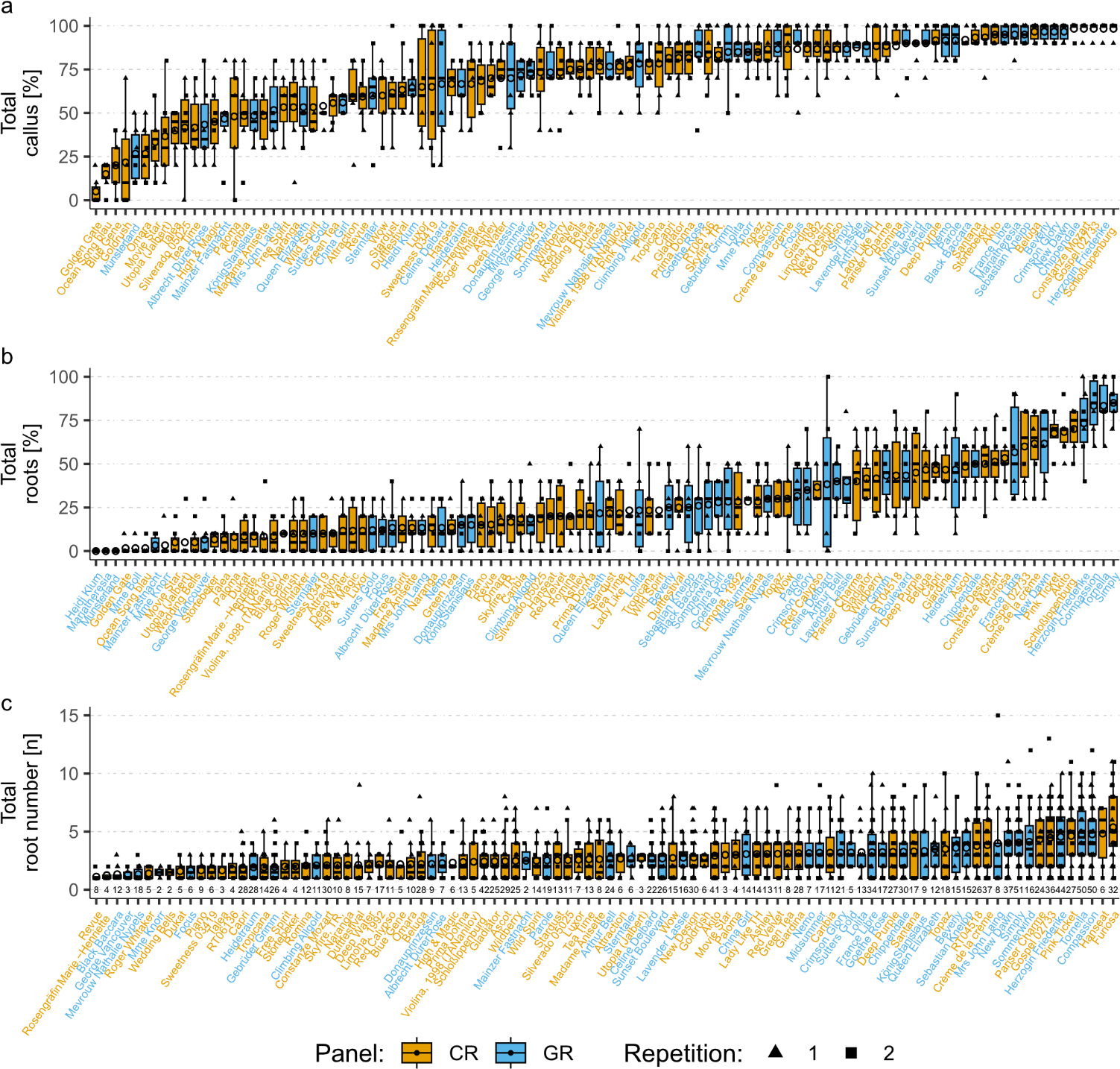


**Fig. S2** Phenotypic data for total callus and root formation from petioles of leaf explants of different rose genotypes. Circles as means, CR and GR representing the cut rose or garden rose panel. 104 genotypes for callus percentage data, 104 genotypes for root percentage data, 98 genotypes for data of root numbers per rooted explant, where only genotypes with at least two rooted explants were considered. N = 3 vessels with 10 explants in each of two repetitions. N for root counting data is mentioned for each genotype below the boxplot

**Table S1** Results of the Variance Component Analysis (VCA) for all traits. Values are percentages of the fractions of the overall variance of the respective traits, rounded to the nearest whole number. NaN = “not a number”; this value couldn’t be computed. - = this factor was not included in the analysis

| **Organ** | **Factors** |  | **Fluo. callus formation** | **Fluo. root formation** | **Fluo. root number** | **Total callus formation** | **Total root formation** | **Total root number** |
| --- | --- | --- | --- | --- | --- | --- | --- | --- |
| Lamina | Total |  | 100% | 100% | 100% | 100% | 100% | 100% |
|  | Genotype |  | 57% | 44% | 9% | 55% | 48% | 9% |
|  | Panel |  | 4% | 5% | NaN | 9% | 7% | 0% |
|  | Experiment |  | 7% | 12% | 2% | 6% | 12% | 5% |
|  | Vessel |  | - | - | NaN | - | - | 2% |
|  | Error |  | 31% | 39% | 89% | 29% | 33% | 84% |
| Petiole | Total |  | 100% | 100% | 100% | 100% | 100% | 100% |
|  | Genotype |  | 54% | 51% | 6% | 56% | 55% | 12% |
|  | Panel |  | NaN | NaN | NaN | 5% | 2% | 1% |
|  | Experiment |  | 8% | 8% | 2% | 3% | 11% | 2% |
|  | Vessel |  | - | - | NaN | - | - | 1% |
|  | Error |  | 37% | 41% | 92% | 36% | 32% | 84% |

**Table S2** Results of the Analysis of Deviance (Type II) for all traits. Given are p values of the Wald chisquare tests. Significant codes: * < 0.05, ** < 0.01, *** < 0.001

| **Organ** | **Factors** |  | **Fluo. callus formation** | **Fluo. root formation** | **Fluo. root number** | **Total callus formation** | **Total root formation** | **Total root number** |
| --- | --- | --- | --- | --- | --- | --- | --- | --- |
| Lamina | Genotype |  | 4.5e-108*** | 1.1e-33*** | 1.2e-03** | 2.3e-131*** | 8.7e-66*** | 2.2e-18*** |
|  | Panel |  | 7.0e-12*** | 1.3e-08*** | 4.1e-01 | 4.0e-17*** | 1.3e-12*** | 2.1e-01 |
|  | Experiment |  | 4.2e-14*** | 3.8e-15*** | 1.0e-01 | 1.4e-17*** | 3.3e-22*** | 1.5e-08*** |
| Petiole | Genotype |  | 5.0e-105*** | 1.0e-45*** | 3.3e-01 | 6.0e-128*** | 7.0e-106*** | 1.2e-39*** |
|  | Panel |  | 5.2e-02 | 8.4e-01 | 7.2e-01 | 1.1e-10*** | 2.3e-02* | 8.1e-02 |
|  | Experiment |  | 4.6e-18*** | 4.1e-12*** | 2.8e-02* | 7.1e-08*** | 1.3e-25*** | 4.2e-04*** |


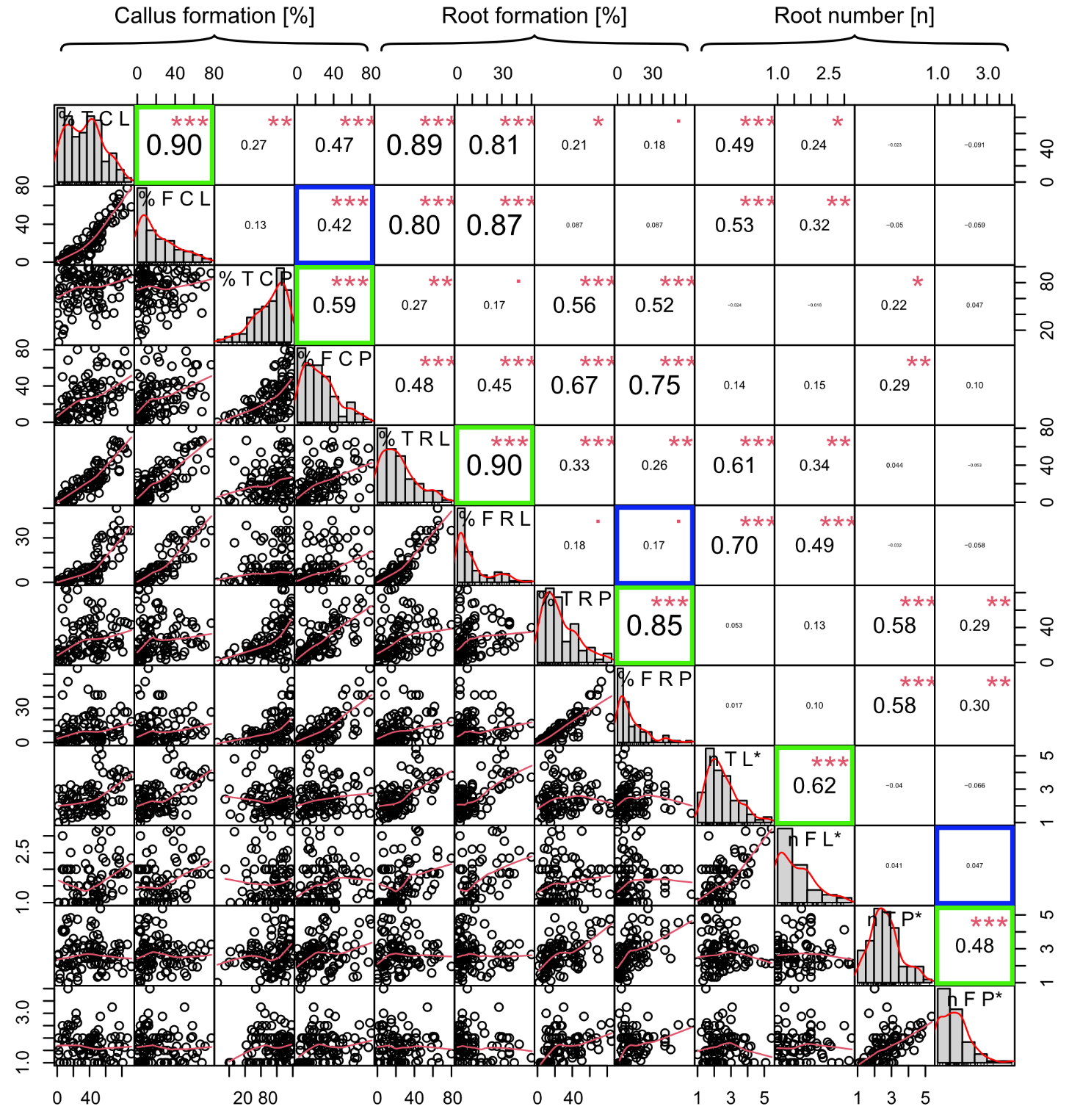


**Fig. S3** Correlations of callus and hairy root formation data. Shown are scatterplot matrix, histograms with kernel density overlays, absolute Pearson correlations and significance asterisks at p = 0.05, 0.01 and 0.001. Highlighted areas: Green marks correlations between data of fluorescent and total callus and root formation. Blue marks the correlations of same fluorescent traits between data from lamina or petiole tissue. Abbreviations: % = percent, T = total, F = fluorescent, C = callus, R = roots, L = lamina, P = petiole, n = number of roots, * = rooted explants


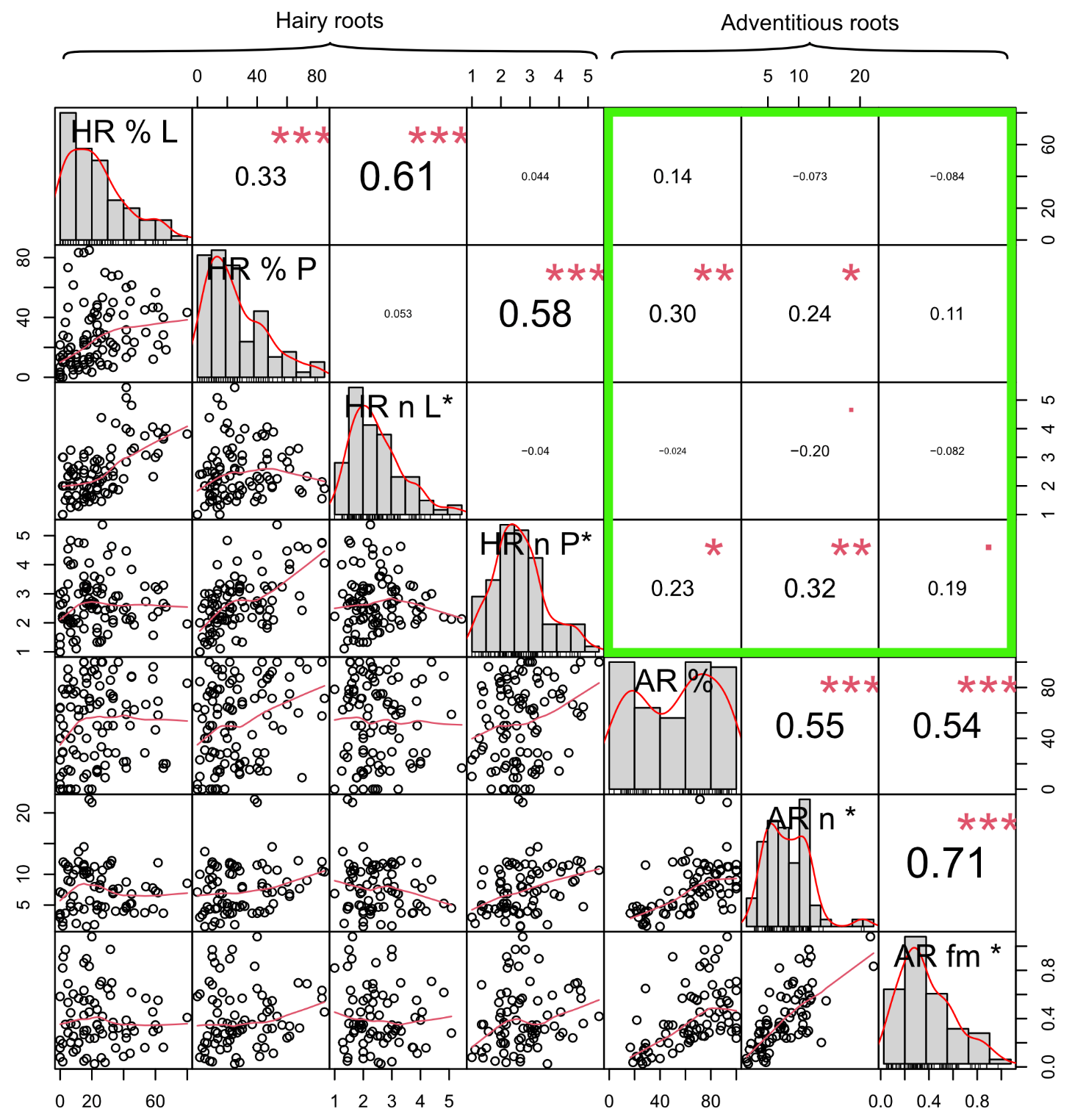


**Fig. S4** Correlations of total root formation data from petioles and laminae and previously published adventitious root formation data of rose cuttings from Wamhoff et al. (2023), using the same 104 genotypes. Shown are scatterplot matrix, histograms with kernel density overlays, absolute correlations and significance asterisks at p = 0.05, 0.01 and 0.001. Highlighted green area: correlation of total HR with AR data. Abbreviations: HR = hairy roots, AR = adventitious roots, % = percent, n = number of roots, L = lamina, P = petiole, fm = fresh mass, * = rooted explants/cuttings


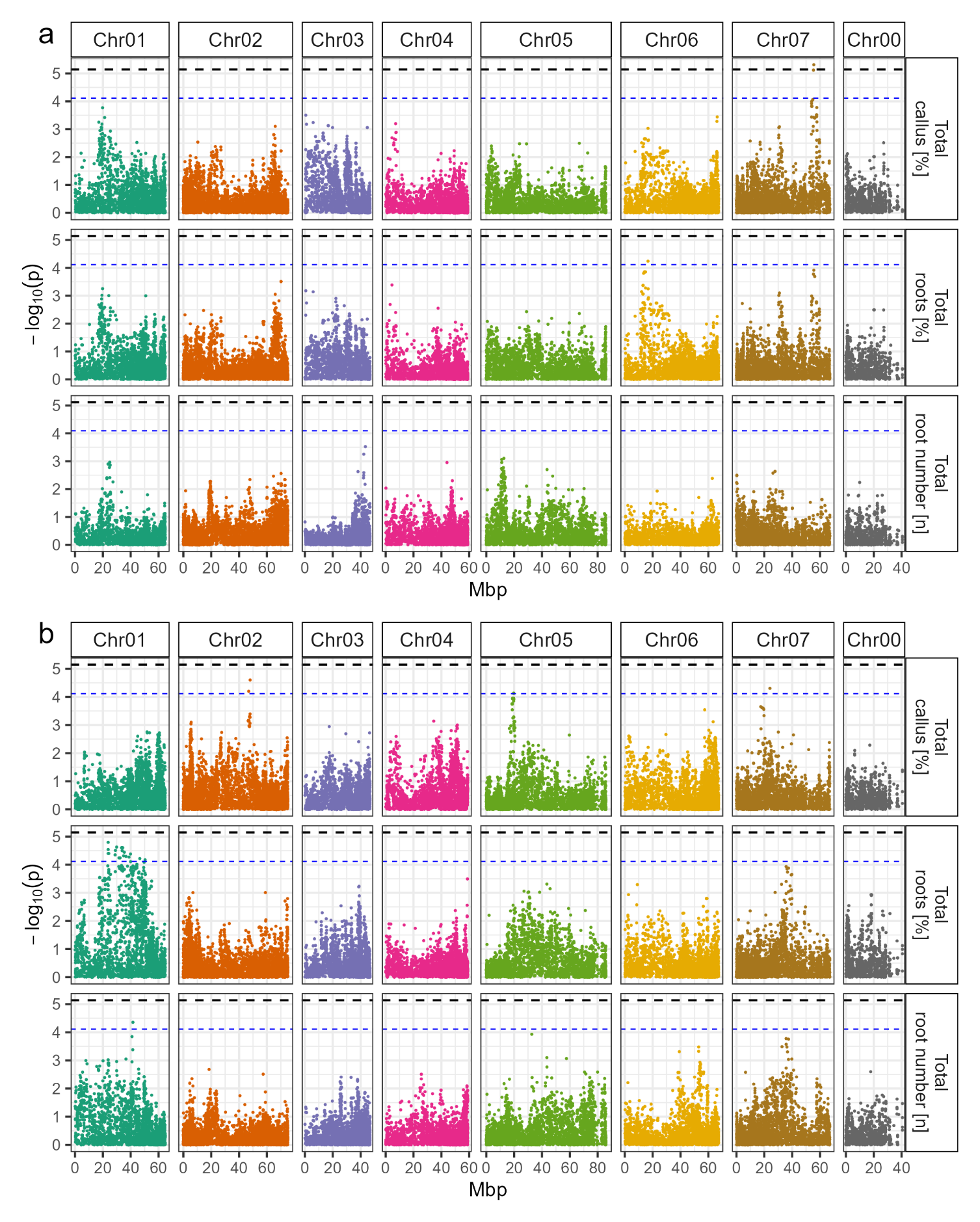


**Fig. S5** Manhattan plots with the results of marker and total trait associations of 23,419 SNPs, which were analyzed with an additive model and shown as –log10 of the SNP´s specific p value. a: Data from laminae. b: Data from petioles. The x-axis shows the positions with respect to the seven Rosa chinensis chromosomes (Hibrand Saint-Oyant et al. 2018) (Chr01-Chr07) in megabase pairs (Mbp). Chr00 covers contigs with SNPs that have not yet been mapped. Root numbers were taken only from root forming explants and genotypes with at least two rooted explants. The horizontal dashed black line indicates the M.eff corrected p value significance threshold, the blue line indicates 80% of the significance threshold

**Table S3** Analysis of peak influence on trait variability. Displayed are single SNPs for each peak with the strongest association, ordered by their position in the genome. Abbreviations: RF% = percentage of root formation, CF% = percentage of callus formation, RN = root number of rooted explants, L = lamina, P = petiole. The partial R^2^ of the total variability is displayed as percentage

| **Peak** | **Trait** | **Marker** | **Chr.** | **Position** | **-log(10)(p)** | **R^2^ fraction [%]** |
| --- | --- | --- | --- | --- | --- | --- |
| 1 | RF% L | Rh12GR_929_1669 | 1 | 21561250 | 3.58 | 12.0 |
| 1 | CF% P | Rh12GR_24085_427 | 1 | 17454693 | 3.82 | 12.9 |
| 2 | RF% P | RhMCRND_17105_1110 | 1 | 36597801 | 6.98 | 23.8 |
| 3 | CF% P | RhK5_1043_2120 | 1 | 48050013 | 3.66 | 12.3 |
| 4 | RN L | RhK5_684_2502 | 2 | 2799617 | 5.12 | 26.9 |
| 5 | CF% L | RhK5_14324_1389 | 7 | 55557365 | 3.72 | 12.5 |

**Table S4** Single SNPs for each peak with the strongest significant difference (effect) between the most diverging AGDs, ordered by their position in the genome. Gene predictions and rose gene IDs were taken from the "Genome Database for Rosaceae" GDR (https://www.rosaceae.org/species/rosa/chinensis/genome_v1.0, last accessed 7th of April, 2024). Abbreviations: RF% = percentage of root formation, CF% = percentage of callus formation, RN = root number of rooted explants, L = lamina, P = petiole

| **Peak** | **Trait** | **Marker** | **Chr.** | **Position** | **-log(10)(p)** | **Effect** | **Rose gene ID** | **Gene prediction** |
| --- | --- | --- | --- | --- | --- | --- | --- | --- |
| 1 | RF% L | RhK5_6877_735 | 1 | 20234077 | 3.47 | 25% | RC1G0151100 | 4'-phosphopantetheinyl transferase superfamily |
| 1 | CF% P | RhK5_5460_1171 | 1 | 18796802 | 3.75 | 37% | RC1G0142000 | FAR1-related sequence 3 |
| 2 | RF% P | no marker found with significant ADG differences | | | | | | |
| 3 | CF% P | RhK5_2022_1767 | 1 | 48067882 | 3.43 | 38% | RC1G0380400 | Embryo defective 1703 |
| 4 | RN L | Rh12GR_47076_193 | 2 | 4777406 | 1.09 | 0.9 roots | RC2G0061800 | Basic helix-loop-helix (bHLH) DNA-binding superfamily protein |
| 5 | CF% L | RhK5_7_1113 | 7 | 57476277 | 1.67 | 23% | RC7G0513100 | Ubiquitin-protein ligase 2 |

References

Hibrand Saint-Oyant L, Ruttink T, Hamama L, Kirov I, Lakhwani D, Zhou NN, Bourke PM, Daccord N, Leus L, Schulz D, van de Geest H, Hesselink T, van Laere K, Debray K, Balzergue S, Thouroude T, Chastellier A, Jeauffre J, Voisine L, Gaillard S, Borm TJA, Arens P, Voorrips RE, Maliepaard C, Neu E, Linde M, Le Paslier MC, Bérard A, Bounon R, Clotault J, Choisne N, Quesneville H, Kawamura K, Aubourg S, Sakr S, Smulders MJM, Schijlen E, Bucher E, Debener T, De Riek J, Foucher F (2018) A high-quality genome sequence of *Rosa chinensis* to elucidate ornamental traits. Nat Plants 4:473–484. https://doi.org/10.1038/s41477-018-0166-1

Wamhoff D, Schulz D, Debener T, Winkelmann T (2023) Genome-wide association study and marker development for adventitious root formation in rose. Acta Hortic 1368:331–340. https://doi.org/10.17660/ActaHortic.2023.1368.42
